# Synthesis of Rosin Acid Starch Catalyzed by Lipase

**DOI:** 10.1155/2014/647068

**Published:** 2014-05-25

**Authors:** Rihui Lin, He Li, Han Long, Jiating Su, Wenqin Huang

**Affiliations:** ^1^Guangxi Key Laboratory of Chemistry and Engineering of Forest Products, College of Marine Sciences and Biotechnology, Guangxi University for Nationalities, Nanning, Guangxi 530006, China; ^2^Nanning Yide Environment Technology Co., Ltd., Nanning, Guangxi 530007, China

## Abstract

Rosin, an abundant raw material from pine trees, was used as a starting material directly for the synthesis of rosin acid starch. The esterification reaction was catalyzed by lipase (Novozym 435) under mild conditions. Based on single factor experimentation, the optimal esterification conditions were obtained as follows: rosin acid/anhydrous glucose unit in the molar ratio 2 : 1, reaction time 4 h at 45°C, and 15% of lipase dosage. The degree of substitution (DS) reaches 0.098. Product from esterification of cassava starch with rosin acid was confirmed by FTIR spectroscopy and iodine coloration analysis. Scanning electron microscopy and X-ray diffraction analysis showed that the morphology and crystallinity of the cassava starch were largely destroyed. Thermogravimetric analysis indicated that thermal stability of rosin acid starch decreased compared with native starch.

## 1. Introduction

With the challenges of fossil resource depletion and the environmental problems, development of polymers from renewable resources either entirely or partially has attracted more and more attention [1]. As a ubiquitous and very abundant biopolymer, starch is considered as the most promising candidate to replace traditional petroleum-based products in many industrial applications [2, 3]. However, as a biodegradable polymer, starch is used very limitedly due to a number of its inherent drawbacks such as poor surface properties, high hydrophilicity, and poor mechanical and thermal properties [4]. Chemical modification such as acetylation, hydroxypropylation, and cross-linking to improve the physicochemical, morphological, thermal, and rheological properties of starches has been developed [5, 6]. In particular, esterification of starch through modifying the hydroxyl groups in the anhydrous glucose unit (AUG) is a common method of chemical modification, and carboxylic acid chains from C4 to C16 have been successfully introduced into starch molecule using fatty acid vinyl esters, fatty acid chlorides, or fatty acid methyl esters as reactants [7, 8]. The esterified products show potential biomedical applications such as carriers for controlled drugs release and other bioactive agents [9]. To produce novel starch ester and broaden its application, much more acid candidates should be investigated for starch ester synthesis.

Rosin acid is the major component (about 90%) of crude rosin which is a kind of natural product obtained from the exudation of conifers. Rosin acid is a mixture consisting primarily of abietic acid, levopimaric acid, and pimaric acid. More than 1 million tons of gum rosin is produced worldwide per year and generally used as ingredients for inks, vanishes, adhesives, cosmetics, medicines, chewing gums, and so forth [10]. Rosin acid attracts much attention in synthesis of new polymeric materials because it is inexpensive, abundant, potentially biodegradable and biocompatible, and capable of chemical modifications due to its special structure [11, 12]. Rosin acid is a mixture of monocarboxylic acids with a characteristic bulky hydrophenanthrene ring structure. The hydrophenanthrene moiety provides rosin acid with substantial hydrophobicity and that facilitates its application in marine antifouling coating materials and in biocides. There are two chemically reactive centers in the rosin acid molecule, the double bond and the carboxyl group [13], and the structural characteristics of rosin acid indicate that it would be a useful reagent for starch modification. The carboxyl group of rosin acid can act as reactive group for esterification reaction and then integrate rosin molecule into native starch as side chains; the bulky hydrophenanthrene group of rosin acid can impart hydrophobicity to the esterified product and alter the thermal properties [14]. In addition, the rosin acid can be converted to a large number of downstream derivatives which means that the rosin acid starch can be further grafted and modified easily, which would lead to novel products with new functionalities [15, 16]. Chemical method has been reported in synthesis of rosin acid starch, and the modified starches show potential applications in waterproof coatings and plastics [14]. However, there are disadvantages in starch ester synthesis by the conventional chemical method; for example, those methods involve harsh reaction conditions and hazardous reagents such as acyl-chlorides.

Lipases are an important group of biotechnological catalysts which catalyze the hydrolysis of triglycerides into free fatty acids and glycerol. Besides, they can also catalyze esterification, acidolysis, interesterification, alcoholysis, and aminolysis and have been widely applied in food, dairy, detergent, and pharmaceutical industries [17]. Lipases have been used as effective catalyst for starch esterification [9], and the enzymatic esterification is superior to the conventional chemical-catalyzed one as it works under milder reaction conditions with higher reaction selectivity and fewer by-products. However, to our knowledge, there has been no report about enzymatic synthesis of rosin acid starch until now.

In this work, rosin acid starch was synthesized successfully using immobilized* Candida antarctica* lipase (Novozym 435) as catalyst. The reaction conditions were investigated, and the structure of rosin acid starch was studied.

## 2. Materials and Methods

### 2.1. Materials

Gum rosin was supplied by Guangxi Wuming Chaoyan Rosin Plant, China and was used directly for esterification reaction. Cassava starch (approximately 17% amylose and 83% amylopectin) was purchased from Guangxi Cenxishi Sanjiao Food Scuffled. Novozym 435 with activity of 10 unit/mg was purchased from Novo Industries, Denmark. DMSO, methanol, and acetone were of analytical grade purchased from Chengdu Kelong Chemical Reagent Co., China.

### 2.2. Esterification of Cassava Starch with Rosin Acid

#### 2.2.1. Pretreatment of Cassava Starch

In order to improve its solubility for the subsequent reaction, the cassava starch was pretreated according to the literature [18]. Cassava starch (4 g) was dissolved in NaOH/urea solution (6 g NaOH and 3 g urea in 100 mL deionized water) completely; then the solution was neutralized with HCl. The starch was washed with 100 mL 95% of ethanol twice after precipitation by the same solvent. Finally, the precipitate was dried at 70°C for 24 h.

#### 2.2.2. General Procedure for Esterification Reaction

Pretreated cassava starch (0.25 g) was dissolved in DMSO (50 mL), followed by the addition of rosin acid at various molar ratios from 1.5 to 7.5 (relative to AUG). To the mixtures, different amount of immobilized lipase ranging from 5% to 25% (m/m, relative to starch) was added. Reaction was carried out at set temperatures for different times. The rosin acid starch was collected after precipitation by adding 150 mL of methanol, then washed with 50 mL methanol twice, and dried at 70°C for 24 h. All reactions were performed in triplicate unless otherwise stated.

### 2.3. Analysis of Reaction Products

#### 2.3.1. Determination of the DS

The DS, defined as the average molar ratio of attached rosin acyl groups per AUG, was determined by a titration method [19]. 1 g sample was dissolved in 50 mL DMSO, then 20 mL 0.2 mol/L NaOH was added, and the mixture was stirred for 4 h at 50°C. Excess NaOH was back-titrated with 0.1 mol/L HCL solution using phenolphthalein as indicator. The DS value of starch ester was calculated as follows:

(1) $$\begin{array}{c} \text{DS}=\frac{162\times C\left(V_{0}-V\right)}{m-285\times C\left(V_{0}-V\right)}, \end{array}$$

where 162 is the molecular weight of the AUG, 285 is the molecular weight of the rosin acid, *V*_0_ and *V* are the titration volume of HCl consumed in native starch and rosin acid starch, respectively, *C* is the molar concentration of HCl, and *m* is the sample mass in dry weight basis.

The reaction efficiency of rosin acid was calculated as follows:

(2) $$\begin{array}{c} \text{CR}=\text{DS}\times \frac{285\times m_{1}}{162\times m_{2}}\times 100\%, \end{array}$$

where *m*_1_ is the starch mass in dry weight basis (g) and *m*_2_ is the rosin acid mass in dry weight basis (g).

#### 2.3.2. FTIR Measurements

FTIR spectra were recorded on a MAGNA-IR 550 spectrometer (Nicolet Instruments Corp., Madison, WI). The samples were mixed with dry KBr at a ratio of 1 : 300. The spectra were recorded in a transmittance mode scanning from 4000 to 500 cm^−1^ with a resolution of 4 cm^−1^.

#### 2.3.3. SEM Analysis

The morphologies of native starch, pretreated starch, and Rosin acid starch were observed using a Supra 55 (Zeiss, Germany) scanning electron microscopy (SEM). Before testing, the samples were mounted onto the specimen stubs with double-sided tape and then coated with a thin layer of gold to make the sample conductive. SEM was performed under high vacuum at an accelerating voltage of 5 kV. The photographs were taken using automatic image capture software.

#### 2.3.4. X-Ray Diffractometry

The X-ray diffraction patterns of the native starch and rosin acid starch samples were measured using a D/MAX 2500 V diffractometer (Rigaku, Tokyo, Japan) under the following conditions: Cu K*α* radiation, Ni filter disk, 30 mA, and 40 kV. The scattering angle (2*θ*) was varied from 4° to 60° with a step width of 0.02°.

#### 2.3.5. Thermal Stability Analysis

The thermogravimetry analysis was carried out in a STA449F3 (NETSCH, Germany) simultaneous thermogravimetry and differential scanning calorimetry (TG/DSC) apparatus. The experiments were performed under argon flow at 30 mL/min and temperature from 28°C to 600°C at a heating rate of 10°C/min.

#### 2.3.6. Iodine Color Analysis

According to literature [20], 0.1% (w/v) sample was colored by iodine solution. The absorbance of the mixture was measured using a UV-VIS spectrophotometer (TU-1901, Beijing Purkinje General Instrument Co., Ltd.).

## 3. Results and Discussion

### 3.1. Synthesis of Rosin Acid Starch

#### 3.1.1. Effect of Substrate Ratio on the DS and the Reaction Efficiency of Rosin Acid

**Figure 1:**
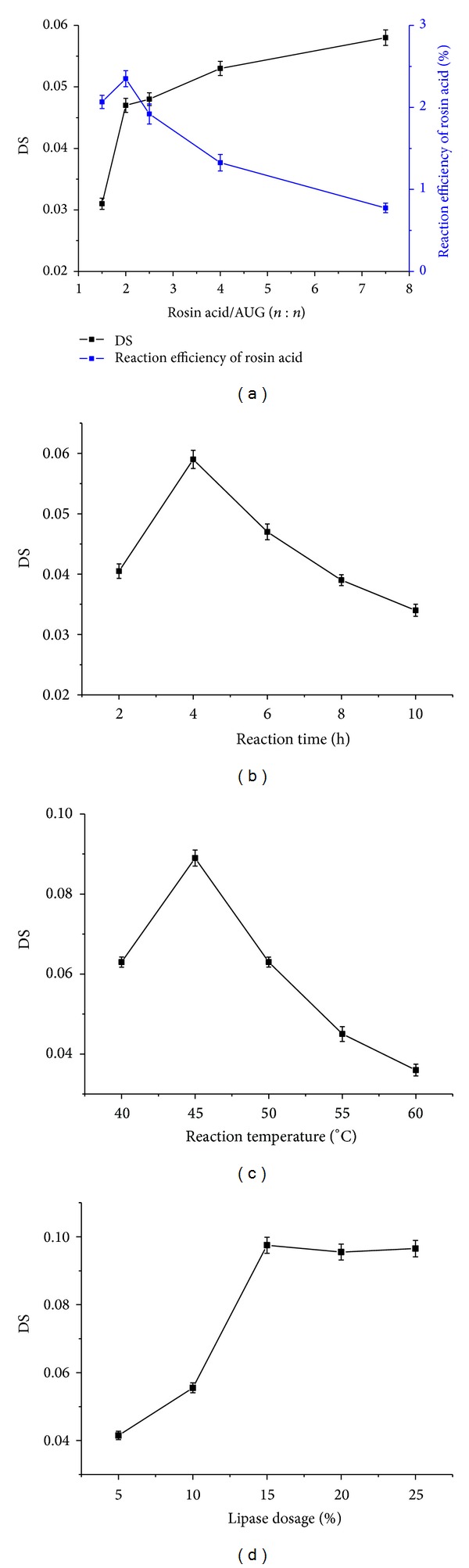
Effect of substrate ratio, reaction time, temperature, and lipase dosage on synthesis of rosin acid starch. All reactions were performed in triplicate, and the mean values were plotted. (a) Effect of substrate ratio on the DS and the reaction efficiency of rosin and other reaction conditions: lipase dosage, 10% (m/m, relative to starch); reaction temperature, 40°C; reaction time, 4 h. (b) Effect of reaction time on the DS and other reaction conditions: molar ratio of rosin acid/AUG, 2 : 1; lipase dosage, 10% (m/m, relative to starch); reaction temperature, 40°C. (c) Effect of reaction temperature on the DS and other reaction conditions: molar ratio of rosin acid/AUG, 2 : 1; lipase dosage, 10% (m/m, relative to starch); reaction time, 4 h. (d) Effect of lipase dosage on the DS and other reaction conditions: molar ratio of rosin acid/AUG, 2 : 1; reaction temperature, 45°C; reaction time, 4 h.

Figure 1(a) suggests that the molar ratio of rosin acid to AUG had an important effect on the product. As can be seen, the DS of rosin acid starch improved remarkably from 0.031 to 0.047, about 51.6% increase, as the molar ratio of rosin acid/AUG increased from 1.5 to 2. However, the promoting role of excess rosin acid on the DS improvement decreased when the ratio exceeded 2. The DS increased to 12.8% as the molar ratio of rosin acid/AUG was raised from 2 to 4, but it just increased to 9.4% as the ratio was raised from 4 to 7.5. Besides, excess rosin acid was unfavorable for the reaction efficiency of rosin acid. As Figure 1(a) shows, the reaction efficiency of rosin acid reached the peak when the molar ratio of rosin acid/AUG was 2, and it decreased sharply as the rosin acid/AUG was raised from 2 to 7.5. Comprehensively considering the DS and the reaction efficiency of rosin in the reaction, the optimal molar ratio of rosin acid to AGU was 2 : 1.

#### 3.1.2. Effect of Reaction Time on the DS

The reaction progress was monitored for 10 h and the result is shown in Figure 1(b). It indicates that the maximum DS value occurred between 2 h and 6 h, and the DS of rosin acid starch changed from 0.041 to 0.059 as the reaction time was increased from 2 to 4 h. However, when the reaction lasted more than 4 h, a gradual reduction of the DS was observed. Similar tendency was also reported by Lu et al., who attempted to synthesize palmitate starch ester using lipase as catalyst [19]. Plausible reasons are that hydrolysis of the starch ester occurred at longer reaction times and/or the enzyme lost some of its activity. Therefore, a reaction time of 4 h was considered as being the most appropriate for the synthesis of rosin acid starch.

#### 3.1.3. Effect of Reaction Temperature on the DS

Reaction temperature was an important factor on DS of the esterification reaction [21]. In the synthesis of rosin acid starch, reaction temperature was set from 40°C to 60°C. As shown in Figure 1(c), a rise of reaction temperature from 40°C to 45°C led to an increase of DS from 0.063 to 0.089. However, DS decreased sharply when the reaction temperature was higher than 50°C, and it dropped to about 0.036 at 60°C. That lipase inactivated under high temperature would be the major reason for this phenomenon [22]. The synthesis of rosin acid starch was carried out with DMSO as solvent, which is polar solvent and will cause activity loss of the enzyme unavoidably. Lu's study in enzymatic esterification starch with palmitate got similar result, but the appropriate reaction temperature rose to 60°C when using ionic liquid mixtures as solvent [19].

#### 3.1.4. Effect of Lipase Dosage on the DS

The degree of esterification was improved by lipase. As shown in Figure 1(d), the DS increased sharply as the dosage of lipase increased from 5% to 15% (in a 4-hour reaction). However, the DS value of rosin acid starch seemed to reach a plateau when the lipase dosage was beyond 15%, which suggested that there was no significant influence on reaction by adding excess amount of lipase under the given conditions. The result indicated that the reaction reached steady state as the DS increased to 0.098 under the experimental condition.

### 3.2. FTIR Analysis

**Figure 2:**
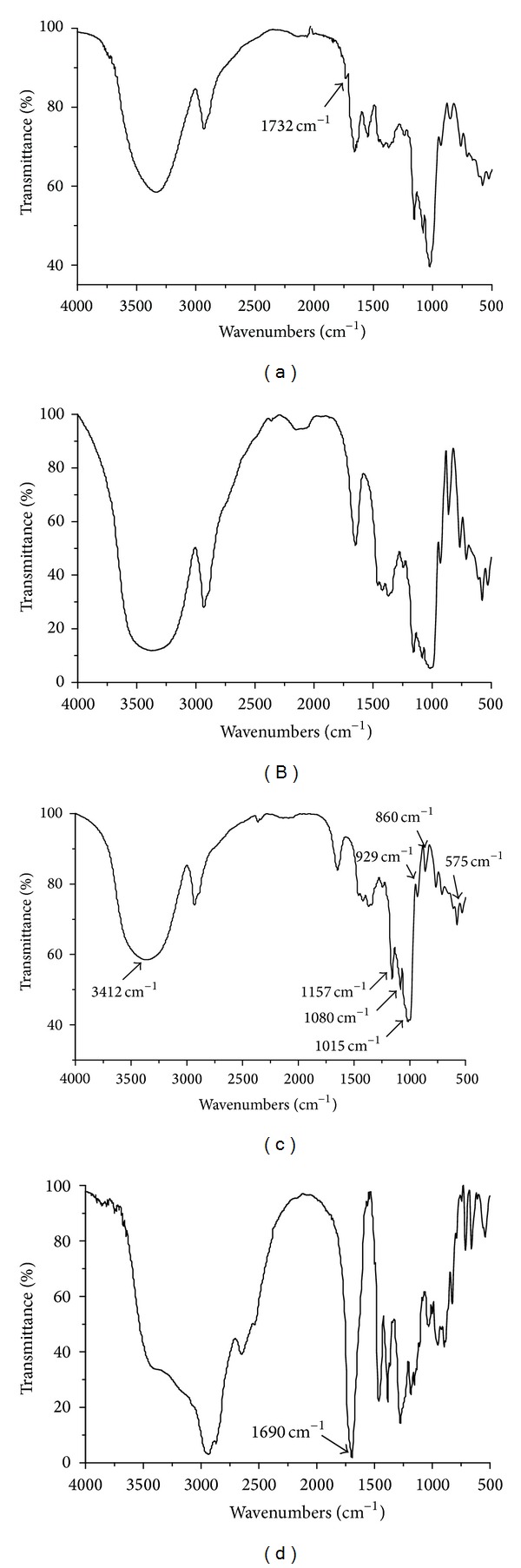
IR spectra of rosin acid starch (a), pretreated starch (b), native starch (c), and rosin (d).

Figure 2 presents the FTIR spectra of the rosin acid starch, pretreated starch, and native starch with rosin acid. It showed that the three starches were similar in peaks at 1157, 1080, and 1015 cm^−1^, which represented the C–O stretching vibrations of AUG, and absorbencies at 929, 860, and 575 cm^−1^ which were attributed to the stretching vibrations of the whole glucose ring [23]. These indicated that the original structure of polysaccharide in starch remained intact after NaOH/urea pretreatment and enzymatic esterification. The peak at 3412 cm^−1^ of the esterified starch, which corresponds to the stretching vibration of OH group [23], decreased in intensity compared with the native starch and the pretreated starch, which indicated that parts of OH group of starch are esterified. In addition, the 1690 cm^−1^ peak corresponding to the carboxyl group [24] in rosin acid molecule disappeared in the spectrum of the esterified starch, but a new peak at 1732 cm^−1^ was observed, which corresponded to C=O stretching in the ester group. It was concluded that rosin acid starch was synthesized successfully.

### 3.3. SEM Microphotographs and XRD Analysis

**Figure 3:**
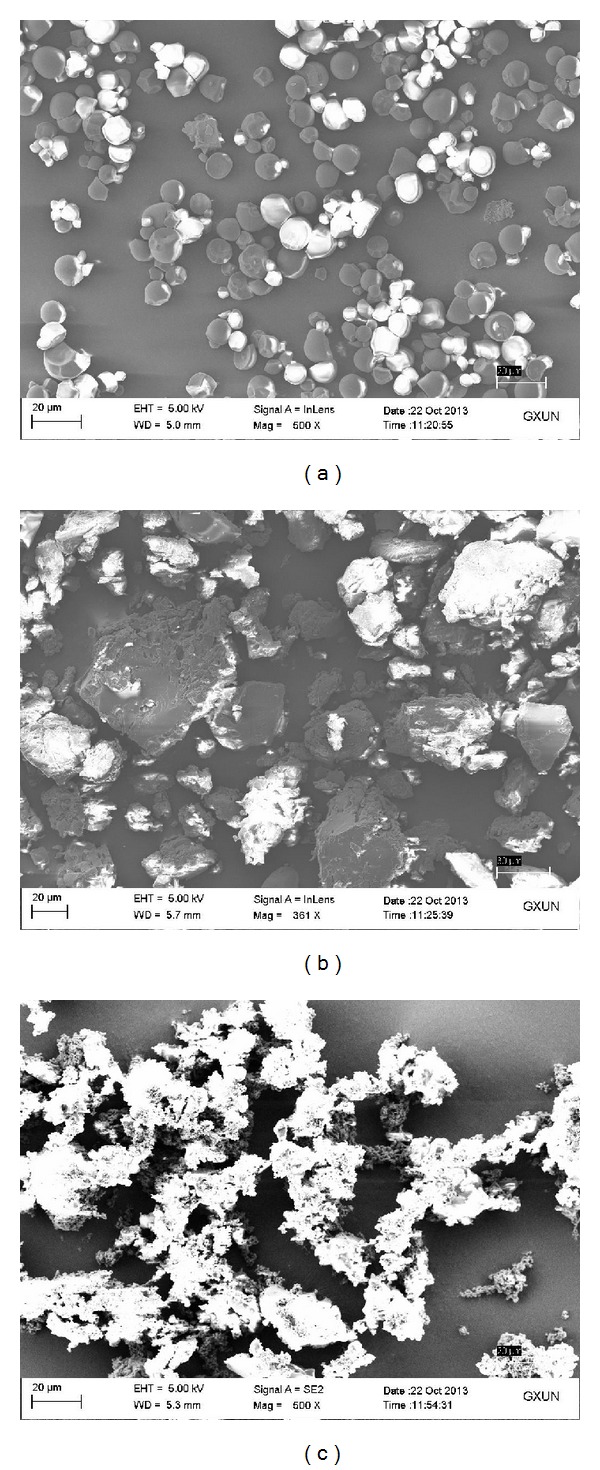
SEM images of native starch (a), pretreated starch (b), and rosin acid starch (c).

**Figure 4:**
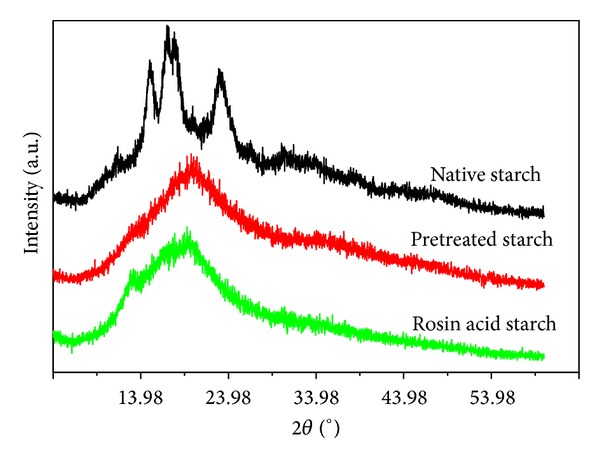
X-ray diffraction patterns of native starch, pretreated starch, and rosin acid starch.

As shown in Figure 3, native starch granules were polygonal or irregular in shape with structurally sound and smooth surface (Figure 3(a)). In agreement with the report of Kasemwong [25], the cassava starch exhibited an A-type crystalline pattern (Figure 4) that showed strong reflections at about 14.98, 16.96, 18.02, and 23.02° and weaker peaks at 11.66, 19.92, 26.72, and 30.46°. NaOH/urea pretreatment caused some changes in the granularity of starch as compared with native starch (Figure 3(b)). The surface of pretreated starch changed from slick to rough but kept some kinds of granule shape. The XRD pattern of pretreated starch showed that there were only dispersive broad peaks. The highly ordered crystalline structure was maintained by the intramolecular or intermolecular hydrogen bonds in native starch [26]. The disappearance of the crystal peak showed that the crystallinity of native starch was damaged during the pretreatment process, which implied that the hydrogen bond of the starch was disrupted by NaOH/urea solution. The rosin acid starch exhibited a whole different morphology compared with native starch and pretreated starch (Figure 3(c)). The figure showed that the starch granules were completely destroyed, losing their individuality and smoothness, exposing the internal laminated structure. Figure 4 shows that rosin acid starch exhibited weak diffraction peaks at 12.74° and 19.32°, which implied that new crystalline regions were formed in rosin starch ester. The phenomenon was similar to the previous reports about crystalline structure of starch acetates [27, 28].

The result of SEM microphotographs and XRD analysis indicated that the starch granules were converted from their semicrystalline structure into amorphous state during the pretreatment and dissolution process, so that the esterification reaction not only occurred in the nonstereotyped area of starch but also occurred inside the crystalline regions. The dissolution process and the integrated rosin acid molecule disrupted the regular hydrogen bond in starch, which prevented the starch from forming its original structure after precipitating from the reaction solution.

### 3.4. Thermal Stability Analysis

**Figure 5:**
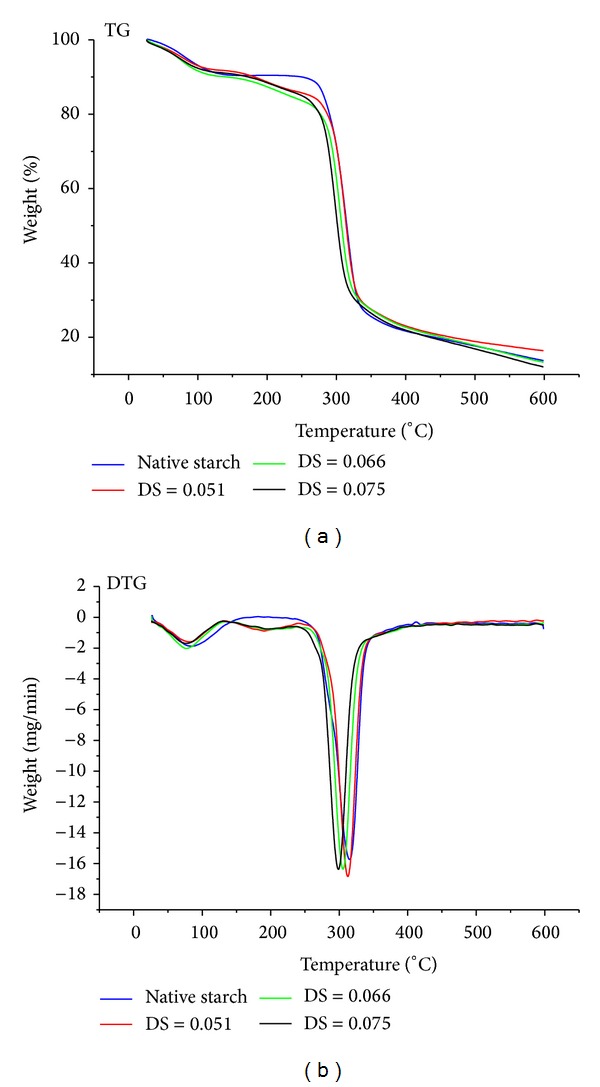
TG and DTG curves of native starch and rosin acid starch with DS of 0.051, 0.065, and 0.075.

The thermal properties of native starch and rosin acid starch were studied by thermogravimetric analysis, and the result is shown in Figure 5. The native starch underwent a two-stage weight loss below 600°C. The first minor one was below 100°C which corresponded to the evaporation of water; the other one began at about 250°C which corresponded to the decomposition of starch. There is a peak in the derivative thermogravimetric (DTG) curve at about 315°C. Based on the DTG curves of esterified starches and native starch, it can be seen that the thermal stability of starch was reduced by esterification with rosin acid. The observed maximum decomposition temperatures for rosin acid starch with DS of 0.051, 0.066, and 0.075 were about 311°C, 303°C, and 297°C, respectively. There were two causes leading to the poor thermal stability of rosin acid starch. First, the esterification process transformed semicrystalline structure of starch granules into an amorphous structure, as shown in SEM and XRD analysis, which would accelerate the thermal decomposition of starch [19]. Secondly, the decomposition peak of major components of rosin acid (including abietic acid, levopimaric acid, and pimaric acid) was about 277°C to 297°C [29, 30], which was lower than the decomposition peak of starch. The weight loss of the rosin acid starch in the region around 300°C was caused not only by decomposition of the starch backbone but also by decomposition of rosin acid molecule grafting onto the starch; the higher the DS was, the lower the decomposition temperature would be. It must be noticed that there was three-stage weight loss for the esterified starches. A new little peak appeared from 142°C to 239°C. The weight loss in this region must be attributed to the evaporation of the impurity substance in gum rosin, such as turpentine, which was very difficult to remove in gum rosin production process [29]. In this experiment, gum rosin was used directly for the rosin acid starch synthesis, so that the impurity substance would be left in the esterified product.

### 3.5. Iodine Color Reaction Analysis

**Figure 6:**
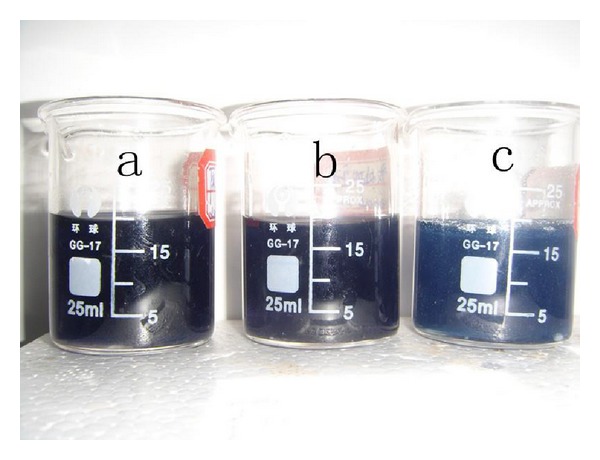
Iodine color reaction with native starch (a), pretreated starch (b), and rosin starch ester (c).

**Figure 7:**
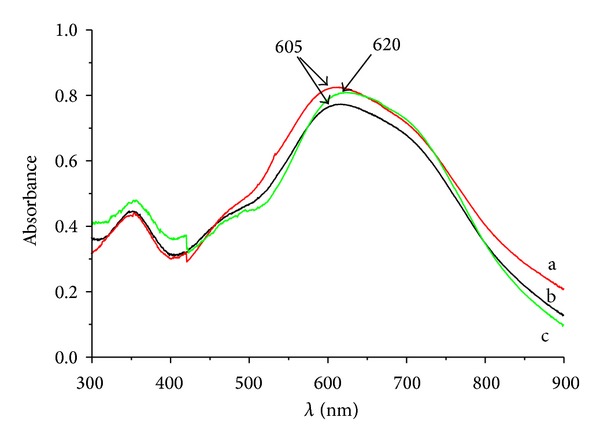
UV-Vis spectra of iodine action on native starch (a), pretreated starch (b), and rosin starch ester (c).

Color complex could be generated by the adsorption action between iodine and starch molecule. The color reaction would change according to the size, shape, and structure of starch [20]. As shown in Figure 6, the iodine adsorption of native and pretreated starch gave purple blue, but the rosin acid starch presented blue. Results of UV-Vis scan indicated that the absorption peak of iodine complex moved to a higher wavelength (from 605 nm to 620 nm) when starch was esterified with rosin acid (Figure 7). The results of iodine color reaction analysis indicated that the structures of native and pretreated starch in water solution were similar, but the structure of rosin acid starch was changed. The structure change in rosin acid starch would not come from the pretreatment process but from the esterification process. The rosin acid was bound to the polysaccharide backbone covalently, and the free hydroxyl group decreased after esterification. All these changes in starch molecule would affect its interaction with iodine and alter the color reaction.

## 4. Conclusion

Rosin acid is a useful reagent for polymers modification because of its special structure, biodegradability, and biocompatibility. We anticipate that esterified starch with rosin acid would not only improve the hydrophobic of native starch but also bring some special properties to the modified product. Then, a lipase catalysis process for the synthesis of rosin acid starch was developed in this work. Results of SEM microphotographs and XRD analysis suggested that pretreatment of starch with NaOH/urea was helpful because it would crash hydrogen bonds in native starch and then make the esterification a homogeneous process. The degree of substitution (DS) 0.098 was achieved in mild reaction conditions after optimizing the molar ratio of rosin acid/AUG, reaction time, reaction temperature, and catalyst dosage. Esterified starch was characterized by FTIR spectroscopy and iodine color reaction analysis. SEM and XRD data indicated that the starch granule was completely destroyed, and some of the crystalline particles became of amorphous state with new crystalline regions formed in the rosin starch ester. Due to the character of rosin acid, the thermal stability of rosin acid starch decreased compared with native starch. In the next step, in order to expand applications of the rosin acid starch, more experiments should be carried out to investigate its properties, such as mechanical properties, biodegradable properties, and the possible antioxidant properties and antibacterial properties.
